# Better you lose than I do: neural networks involved in winning and losing in a real time strictly competitive game

**DOI:** 10.1038/srep11017

**Published:** 2015-06-05

**Authors:** Mikhail Votinov, Juergen Pripfl, Christian Windischberger, Uta Sailer, Claus Lamm

**Affiliations:** 1 Social, Cognitive and Affective Neuroscience Unit, Department of Basic Psychological Research and Research Methods, Faculty of Psychology, University of Vienna, Liebiggasse 5, A-1010 Vienna, Austria; 2 MR Center of Excellence, Center for Medical Physics and Biomedical Engineering, Medical University of Vienna, Lazarettgasse 14, A-1090, Vienna, Austria; 3 Department of Psychology, University of Gothenburg, PO Box 500, SE-405 30 Gothenburg, Sweden; 4 Dept. of Psychiatry, Psychotherapy and Psychosomatics, RWTH Aachen University, Pauwelsstr. 30, 52074, Aachen, Germany

## Abstract

Many situations in daily life require competing with others for the same goal. In this case, the joy of winning is tied to the fact that the rival suffers. In this fMRI study participants played a competitive game against another player, in which every trial had opposite consequences for the two players (i.e., if one player won, the other lost, or vice versa). Our main aim was to disentangle brain activation for two different types of winning. Participants could either win a trial in a way that it increased their payoff; or they could win a trial in a way that it incurred a monetary loss to their opponent. Two distinct brain networks were engaged in these two types of winning. Wins with a monetary gain activated the ventromedial prefrontal cortex, an area associated with the processing of rewards. In contrast, avoidance of loss/other-related monetary loss evoked activation in areas related to mentalizing, such as the temporo-parietal junction and precuneus. However, both types of winnings shared activation in the striatum. Our findings extend recent evidence from neuroeconomics by suggesting that we consider our conspecifics’ payoff even when we directly compete with them.

Competition between and among different living forms is one of the most important forces in evolution, and human beings are no exception. We compete for food, territory, mates, and also in all possible areas of our social life, like sports, politics, education and business. The more a certain resource is limited, the more competition arises between individuals. In the field of game theory, a mathematical model describes one type of competitive situation, called “zero-sum” game or *strictly competitive* game, in which one participant’s gains are the result of equivalent losses for the adversary^1^. In this type of game, the net change in total wealth allocated to all participants is zero, because in each round the available wealth is allocated to one participant at the expense of the other. Good examples of this type of competition are gambling and sport contests. These types of competitions usually involve winners and losers and a relationship where the win of one competitor signifies a loss to the other. This setup implies that participants directly benefit from the other’s misfortune.

Several neuroimaging studies have shown that activation in “mentalizing” brain networks including medial prefrontal cortex (MPFC), temporoparietal junction (TPJ) and temporal pole (TP) are involved in competition^2^,^3^,^4^,^5^. In particular, distinct regions were found to be selectively associated with cooperation and competition, notably the orbitofrontal cortex in the former and the inferior parietal and medial prefrontal cortices (MPFC) in the latter^2^. In addition, activation in bilateral TPJ was associated with competition during a bargaining game^3^, and activations in the MPFC, TPJ, right fusiform gyrus and TP were associated with the opponent’s response in a competitive domino game^4^. Other competition studies demonstrated engagement of the ventral striatum (VS) and the ventromedial prefrontal cortex (VMPFC), brain areas associated with the “reward” brain network^6^,^7^,^8^.

However, there is a lack of neuroimaging studies which investigated strictly competitive games, i.e., games in which decisions affect both competitors (see however^9^,^10^ for related earlier work). Therefore, the goal of the current study was to disentangle evoked neural responses for different types of self-related and other-related monetary feedback during a real time strictly competitive game, in which each participant’s move had direct consequences both for himself/herself and the opponent.

Compared to previous studies, our experimental design enabled investigating two different types of wins and losses. Participants could either win against another participant by gaining the money at stake in that round, instead of the opponent, whose payoff in turn would be zero; or they could win by averting a monetary loss, in which case, however, the opponent would suffer from a monetary loss. Hence, there were also two mirrored types of losses for participants. One was losing without monetary consequences, but with the opponent winning money. Another one was a monetary loss, in which case the opponent won the trial, but without an increase of money. For this purpose, we implemented a simple reaction time task in the form of a modified monetary incentive delay (MID) task^11^ in which two participants (one in the scanner, one outside) competed to respond faster than the other in “gain” and “loss” trials, to either gain the money of that round, or to avoid its loss. We measured brain responses to the outcome of this game, i.e., during the feedback phase in which participants were informed who had responded faster in that round, and hence had won the trial.

Based on previously published fMRI studies which investigated competition and reward processing, we predicted that winning in the game would engage different brain networks, depending on the type of winning. We expected that a monetary gain trial would results in higher activation in reward processing areas, while trials in which winning incurred losses to the opponent would reveal activation in “mentalizing” areas because of an implicit coding of the aversive response of the opponent due to his or her monetary loss^6^,^12^,^13^,^14^,^15^.

Furthermore, we predicted that self-related monetary losses in the game would reveal activation in the insulae, medial/anterior cingulate cortex (M/ACC), and lateral prefrontal cortex, as these areas have been associated with pain, monetary loss, and punishment in previous research^6^,^16^,^17^,^18^,^19^.

Since competition in this game conceptually resembles a social interaction, we also wanted to investigate the involvement of brain structures related to the self-other distinction during the real-time strictly competitive game. More specifically, we wanted to explore which brain areas are involved in processing the feedback for the two different types of wins and losses, and clarify whether these brain areas are overlapping or distinct.

## Results

### Behavioral data

The ANOVA revealed a main effect of the factor outcome (F(2,136) = 54.16, p < 0.001, η^2^ = 0.44). Post-hoc tests demonstrated that there was no significant difference in reaction time between gain (mean ± SD ; 198 ms ± 22) and loss cue trials (197 ms ± 21, p = 1), while both gain and loss cue trials resulted in faster reaction times than neutral cue trials (216 ms ± 17; p < 0.001 for both comparisons).

### *fMRI* data

**Figure 1:**
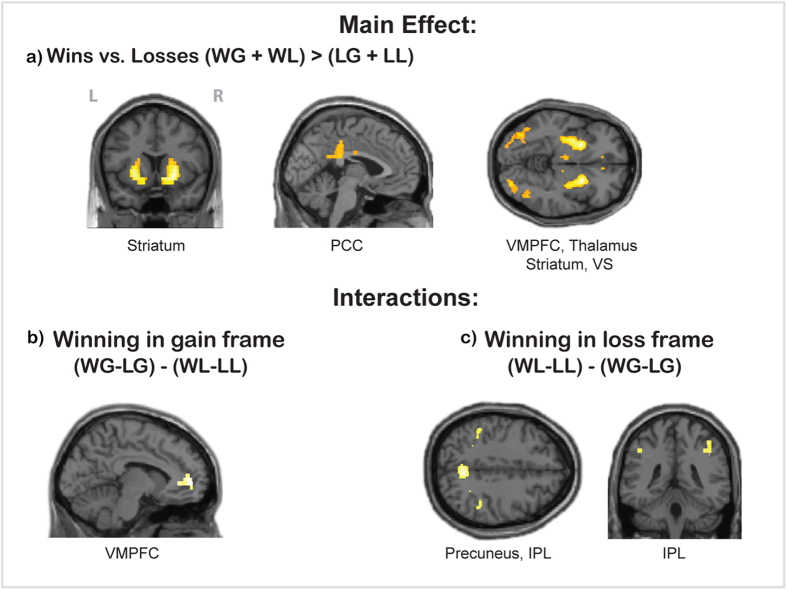
Whole brain activation of all participants for contrasts: **a**) main effect of wins (WG[3:0] + WL[0:−3]) > (GL[0:3] + LL[−3:0]) revealed activation in bilateral striatum, VMPFC, PCC, thalamus, and occipital (visual) areas (threshold p < 0.05 FWE corrected at voxel level); **b**) interaction contrast winning in gain frame versus winning in loss frame (WG[3:0] − LG[0:3]) > (WL[0:−3] − LL[−3:0]) revealed activation in VMPFC (threshold p < 0.05 FWE corrected at cluster level); **c**) interaction contrast winning in loss frame versus winning in gain frame (WL[0:−3] − LL[−3:0]) > (WG[3:0] − LG[0:3]) revealed activation in left IFG, bilateral temporo-parietal lobe and precuneus. The threshold is P < 0.05 FWE corrected, at voxel level.

The main effect of *wins* (WG[3:0] + WL[0:−3]) > (LG[0:3] + LL[−3:0]) revealed activation in bilateral striatum, VMPFC, PCC, thalamus, left OFC and occipital (visual) areas (Fig. 1a). The main effect of *losses* (LG[0:3] + LL[−3:0]) > (WG[3:0] + WL[0:−3]) did not reveal any significant activation.

The interaction contrast, with the participant’s monetary payoff being positive, (i.e., (WG[3:0] − LG[0:3]) > (WL[0:−3] − LL[−3:0]), did not reveal activation using the more stringent voxel-wise multiple comparison correction threshold. However, using a slightly more liberal cluster level correction threshold (P = 0.05, cluster selection intensity threshold of P = 0.001) revealed activation in VMPFC (Fig. 1b). Another interaction contrast, which represented non-monetary winning (WL[0:−3] − LL[−3:0]) > (WG[3:0] − LG[0:3]), revealed activation in the temporo-parietal lobe and precuneus (Fig. 1c).

**Figure 2:**
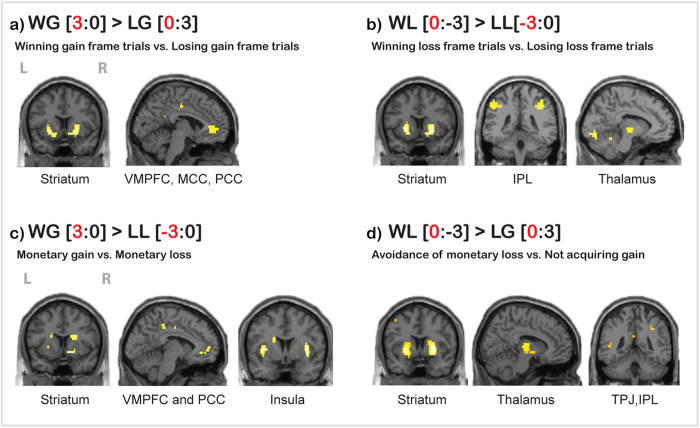
Whole brain activation of all participants for: **a**) winning in gain frame trials (WG[3:0] > LG[0:3]) revealed activation in bilateral striatum, VMPFC, MCC and PCC; **b**) winning in loss frame trials (WL[0:−3] > LL[−3:0]) revealed activation in **b**ilateral striatum, bilateral temporo-parietal lobe, thalamus, and occipital (visual) areas; **c**) monetary gain versus monetary loss (WG[3:0] > LL[−3:0]) revealed activation in bilateral striatum, VMPFC, PCC and bilateral middle insulae; d) avoidance of monetary loss/opponent punishment versus missed/not acquiring the monetary gain (WL[0:−3] > LG [0:3]) revealed activation in bilateral striatum, thalamus, TPJ and temporo-parietal lobe. The threshold is P < 0.05 FWE corrected, at voxel level. *Note:* L/R = left/right side of the brain.

The contrast (WG[3:0] > LG[0:3]), which compared winning and losing in Gain frame trials revealed activation in bilateral striatum, VMPFC, MCC and PCC (Fig. 2a). Conversely, the contrast (WL[0:−3] > LL[−3:0]), which compared winning and losing in Loss frame trials demonstrated activation in inferior parietal lobule (IPL), thalamus, bilateral striatum, and visual cortex (Fig. 2b). However, we did not observe activation for the losing conditions, i.e., neither for the contrast LG[0:3] > WG[3:0] nor for LL[−3:0]) > WL[0:−3].

The contrast WG[3:0] > LL[−3:0] , where we compared winning with monetary gain versus losing with a monetary loss demonstrated greater activation in bilateral striatum, middle/posterior bilateral insula, VMPFC, medial prefrontal cortex (BA10), ACC, PCC, lateral OFC, and temporal pole (TP) (Fig. 2c; Table 1).

The comparison LL[−3:0] > WG[3:0] did not reveal significant activation. Only after reducing the threshold to 0.001 uncorrected we observed activation in bilateral IFG, TPJ and precuneus.

The next step was to investigate brain activation for the processing of the other type of winning. The contrast WL[0:−3] > LG[0:3] revealed activation in bilateral striatum, bilateral inferior frontal gyrus (IFG), inferior parietal lobule (IPL), TPJ, PCC, precuneus, thalamus and midbrain (Fig. 2d, Table 2).

However, the opposite contrast LG[0:3] > WL[0:−3] did not reveal any activation. When reducing the threshold to P = 0.001 (uncorrected for multiple comparisons), however, we observed activation in bilateral middle insulae and left hippocampus.

### Conjunction analysis

**Figure 3:**
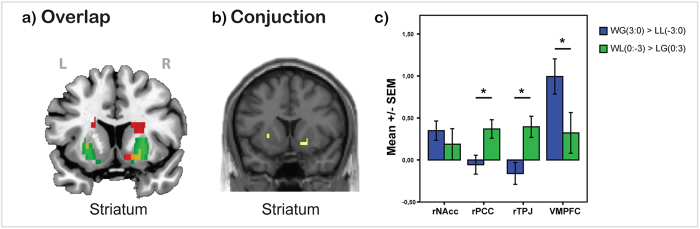
Analysis of the contrasts, which represent two types of winning (WG[3:0] > LL[−3:0]) and (WL[0:−3] > LG [0:3]). **a**) Overlap of activation for both contrasts in striatum, where red color is activation for WG[3:0] > LL[−3:0], green color is activation for WL[0:−3] > LG [0:3] and yellow color is overlap of activation from both contrasts; **b**) Conjunction analysis of both contrasts revealed activation only in striatum. The threshold is P < 0.05 FWE corrected, at voxel level; **c**) BOLD signal (Parameter estimates ± SEM) from rNAcc, rPCC, rTPJ and VMPFC. Contrast GW[3:0] > LL[−3:0] demonstrated significantly higher activation in VMPFC, but significantly lower in rTPJ and rPPC then WL[0:−3] > LG[0:3] contrast.

The conjunction analysis for both winning contrasts (WG[3:0] > LL[−3:0] and WL[0:−3] > GL[0:3]) revealed that only the striatum showed overlapping activation in both contrasts (Fig. 3(a,b), Table 3).

### ROI analysis

Repeated measures ANOVA revealed a significant main effect of ROIs (F(1,2.17) = 8.7, p < 0.001, η^2^ = 0.06) and a ROIs*Contrast interaction (F(1,2.17) = 11.39, p < 0.001, η^2^ = 0.077). Bonferroni-corrected post-hoc comparisons demonstrated significantly higher activation for the GW[3:0] > LL[−3:0] contrast then for the WL[0:−3] > LG[0:3] contrast in VMPFC (0.99 ± 0.2; 0.32 ± 0.22, respectively; p = 0.038), but significantly lower activation in the rTPJ and the rPPC (rTPJ : −0.16 ± 0.12; 0.39 ± 0.12, p = 0.002; rPPC: −0.06 ± 0.11; 0.37 ± 0.11, p = 0.008). However there was no significant difference in the rNAcc (0.34 ± 0.15; 0.19 ± 0.15, p = 0.46) between the contrasts (Fig. 3c).

## Discussion

The current study investigated brain activation in a strictly competitive game, in which the participant’s performance had direct consequences for the outcome of the other player in the game. We particularly targeted two different types of winning in such game: Winning with monetary gain and winning without a gain, but incurring a monetary loss to the opponent. Winning trials with monetary gain of the participant were associated with increased activity in the bilateral striatum, VMPFC and middle/posterior insula. Winning which avoided a monetary loss to the participant, but was also associated with a monetary loss of the opponent, also engaged the bilateral striatum. In addition, it activated the inferior frontal gyrus (IFG), inferior parietal lobe (IPL), and precuneus. The behavioral data demonstrated that participants were faster in reaction time to monetary cues comparing to non-monetary ones, which is in line with other studies which used similar task^20^,^21^. Before we interpret these findings in more detail, we will discuss the main effects and interaction results of our design.

### Main effect and interactions

The main effect of *winns* revealed, as expected, activation in regions like bilateral striatum and VMPFC, which is in line with many studies associating these areas with positive valence, in particular in the context of economic decision making (see^12^ for recent meta-analysis). The interaction contrasts with self-related monetary winning (in gain frame trials) and self-related non-monetary winning (in loss frame trials) revealed activation in distinct areas. The former showed activation in VMPFC (at a cluster level correction threshold), whereas the latter indicated stronger engagement of the bilateral temporo-parietal lobe and precuneus. Overall, these results demonstrate that distinct regions were involved in the two types of winning.

The winning condition associated with monetary reward engaged more VMPFC, while the winning condition associated with avoidance of monetary loss/opponent’s punishment involved the temporo-parietal region. As stated above, VMPFC engagement was repeatedly observed in numerous studies investigating primary and secondary rewards, and value computation. Thus, the increasing activation in this region when subjects increased their own monetary outcome is in line with the previous literature.

The activation in precuneus and temporo-parietal areas can be explained based on findings from social neuroscience associating these areas with empathy, mentalizing and theory of mind (for review see^22^,^23^). Engagement of these functions might be related to the fact that in the monetary loss trials, subjects necessarily had to incur a loss in their opponents, in order to avoid a monetary loss in themselves. Therefore, activation in these areas may be related to a higher propensity to feel with or to adopt the perspective of the opponent’s negative outcome in such a setting.

Notably, previous studies have also identified MPFC to play a role in mentalizing, while this area was not activated in our study. However, a recent meta-analysis of mentalizing/theory of mind studies suggests that mPFC is predominantly related to trait and false belief statements, which might explain the lack of activation in the present setting which required state inferences^24^.

Since the focus of this work was also to investigate responses to different types of wins and losses, we will therefore now discuss the specific results of the corresponding contrasts in detail.

### Monetary gain WG[3:0] > LL[−3:0]

The analysis of different types of winning showed that own monetary gains revealed, as expected brain activation in VS, VMPFC and PCC. This confirms the results of numerous studies investigating the processing of primary and secondary rewards in both social and non-social contexts (for reviews see^12^,^25^,^26^,^27^).

Additionally, mPFC and ACC also showed activation when a monetary gain was received. It has been suggested that these areas represent self-perception or self-knowledge in social contexts, as well as the ability to differentiate the self from other objects, and to recognize attributes and preferences related to oneself^15^,^28^,^29^.

### Losses (LL[−3:0] > WG[3:0]) and (LG[0:3] > WL[0:−3]) contrasts

Contrary to our hypothesis, we did not observe activation for the contrast *LL[*−3*:0]* *>* *WG[3:0]* in the insula and in the ACC for self-related monetary loss. Only after reducing the threshold to 0.001 uncorrected we observed activation in bilateral IFG, TPJ and precuneus. A similar situation was given with another losing contrast LG[0:3] > WL[0:−3], where we observed activation in bilateral insulae and left hippocampus only after reducing the threshold to 0.001 uncorrected. One explanation for a lack of significant activity for these contrasts is that the numbers of loss trials was lower than the win trials, to make the task settings believable and let the subjects get more profit. This might however have reduced the statistical power of analyses targeting higher activation in the loss trials.

### Avoidance of monetary loss /opponent’s punishment WL[0:−3] > LG[0:3]

This contrast aimed to compare a situation in which participants achieved a zero payoff, but avoided a monetary loss which instead was incurred to the opponent, with an equivalent situation in terms of payoff, which however carried a monetary gain for the opponent. Interestingly, this revealed rather distinct neural networks. Additionally to activation in VS and PPC, we observed activation in temporo-parietal areas like bilateral inferior parietal lobule, TPJ, TP, precuneus, and the IFG. The temporo-parietal areas are often described as empathy- and mentalizing-related areas that are recruited when individuals need to understand and predict other people’s intentions and beliefs^14^,^30^,^31^,^32^. This is particularly important in social contexts like competition, where we need to observe or are directly made aware of how our own actions and their outcomes affect others.

Neuroimaging studies which used other types of competition tasks also confirmed an engagement of these areas. For example, competition was associated with activation in the right IFG, bilateral temporal lobe, bilateral fusiform and bilateral precuneus during an adapted Stroop Task^33^. Competition was also associated with activation in the inferior, parietal and medial prefrontal cortices^2^. Two more studies observed competition-related brain activation in TPJ and TP during a competitive ultimatum game^3^ and a competitive domino game^4^. A recent study by Radke *et al.* also demonstrated activation in parietal cortices and TP when the action of a participant in the game had negative consequences for their opponents^5^.

In addition to temporo-parietal activation during other’s-related monetary loss, we observed lateral prefrontal (LPFC) and bilateral IFG activation. Earlier accounts had associated these areas with distinguishing self from other^30^. More recently, several neuroimaging studies observed activation of IFG during loss aversion^34^,^35^, safe reward^36^ and risk aversion^37^. Hence, activation in IFG during observing someone else’s misfortune might represent a general mechanism of processing losses.

### ROI results

Our exploratory ROI analysis demonstrated that VMPFC was activated significantly higher during *Monetary gain* contrast than during *Avoidance of monetary loss/opponent’s punishment* contrast, while activation for right PCC and right TPJ showed the opposite pattern of activation. These findings also support our hypothesis about that different brain networks involved depend on the type of winning. The coordinates for rTPJ and rPCC were taken from the peak of activation of the contrast “Mentalizing about Others versus Mentalizing about Yourself” from the study of^38^ and we observed higher activation in these regions for the contrast where the participant had the stronger negative impact on the payoff for the opponent.

### Shared activation for own monetary gain and avoidance/other monetary loss

One explanation for shared activation in the ventral striatum when participants won a trial and received a monetary gain, and when winning in loss frame trials (which incurred a loss to the opponent) is that this activation is associated with general reward processing^39^. According to a recent concept it may indicate an enhanced motivational value in the form of incentive salience attribution to stimuli perceived at that moment^40^. In addition, in our ROI analysis there was no difference in activation in right nucleus accumbens for different types of winning. This also suggests that activation in VS is associated with generalized aspects of winning.

It might be argued that our results can also be explained in a prediction error framework, as areas such as the striatum have been associated in the coding of prediction errors^41^. However, the present design was not tailored to analyze or interpret our results within such a framework. This is so because the outcome of each trial was very ambiguous and hard to predict for the player, which likely resulted in a complex, subjective and individually varied mixture of positive and negative expectations and expectation violations which could not be modeled.

A different explanation is that a competitive situation may elicit different types of emotional reactions. Participants may experience empathy while observing failure of a group member, but failure of a rival may cause *Schadenfreude*, i.e. pleasure about someone else’s misfortune. One possible condition when *Schadenfreude* may arise is when people can gain from another’s misfortune^42^. Takahashi *et al.* (2009) demonstrated a stronger correlation between activation in ventral striatum and self-reported *Schadenfreude* in a situation when misfortunes happened to envied persons^43^, and a different study concluded that the striatum plays a role in mediating the emotional consequences of social comparison during competition^8^. Furthermore, in a social group competition an increase of VS activation was observed during success of the favored team or failure of the rival team, even against a third team^6^. Similarly, the VS was activated during watching a negatively evaluated out-group member receiving pain^44^, and observing others making errors^13^.

Although we did not explicitly measure the level of *Schadenfreude*, pleasantness and motivation in this study, we speculate that activation in the striatum partially is related to these aspects. This interpretation is further supported by a recent study which showed that participants’ self-evaluations of pleasantness were associated with activation in the VS when winning in a competitive game^45^. Additional research is needed to directly examine the link between VS with *Schadenfreude* and motivation during competitive interactions.

However, we need to take into account that humans are not exclusively motivated by material self-interests, but that people often also care for the well-being of others^46^. Moreover it was found that individual differences in prosocial value orientation are important for the allocation of recourses between self and others, and that amygdala, striatum and VMPFC play a critical role mediating this effect^47^,^48^,^49^ . Our task design provided no choice but to punish the opponent in the loss condition, and this certainly has affected subjects with differences in prosocial orientations in a different way. Since we however did not collect data on individual differences in prosocial orientation, the question how the neural networks identified in our study are related to such differences needs to be clarified by future studies.

Taken together, this study demonstrates that two distinct brain networks are engaged when people process of two types of winning in the game, i.e., own monetary gain and others-monetary loss. A medial-frontal network demonstrated activation for own monetary gain, while a temporo-parietal network was more involved in response to others’ monetary losses. Both types of winning in the game shared activation in the VS which may represent the “joy of winning” for outperforming someone else during competition. Alternatively, this may suggest that the misfortunes of opponents were treated as reward and elicited *Schadenfreude*.

In conclusion, the present study demonstrated that, depending on the type of winning in the competitive game, distinct brain areas are engaged in the processing. Our results provide new insights for understanding brain function during competitive contexts and fundamental features of human social interactions.

## Methods

### Participants

Sixty nine healthy volunteers (38 females and 31 males) participated in the experiment. The average age was (mean ± SD) 23.8 ± 5.4 years old. All volunteers had no history of psychiatric or neurological disorders or contraindications for high-field MRI scanning. All were right-handed as assessed by the Edinburgh Handedness Inventory. All participants signed informed consent before the study and the study protocol was approved by the ethics committee of the Medical University of Vienna. The methods were carried out in accordance with approved guidelines.

### Competitive Task design

We employed a Competitive Incentive Delay (CID) task, which was a modification of the Monetary Incentive Delay (MID) task introduced by Knutson and colleagues^11^. The CID differed from the MID only by the fact that participants played against another person, rather than trying to stay within a pre-set reaction time as in the original MID.

More specifically, participants were told that they were competing with another participant, to whom they were connected via the computer network. In reality, though, they were playing “against” a pre-programmed computer algorithm. To make the task more believable, all participants had taken part in practice trials, together with the experimenter and before entering the scanner. In these practice trials, experimenter and participant played the CID against each other in real time and while sitting next to each other, in front of a computer. The practice trials also served to familiarize the participants with the task and to minimize learning effects during the experiment. After entering the scanner and before the task started, the abstract silhouette of an opponent and a message that the connection with the opponent’s computer had been initiated was shown on the screen. Participants did not get any personal information about their adversary. In reality, they played against a pre-set computer algorithm, and were debriefed after completion of the experiment.

**Figure 4:**
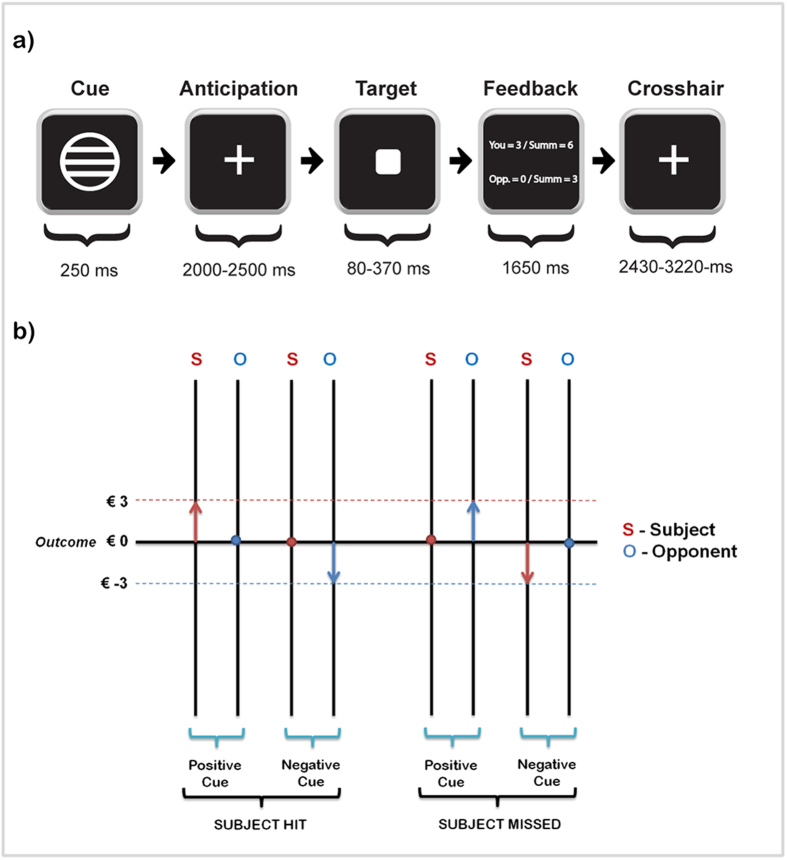
a) Schematic illustration of one trial of the competitive incentive delay (CID) task performed by subjects in the MRI scanner; b) Schematic illustration of monetary outcomes in situations when participants reacted faster or not than the opponent, in gain and loss frames.

The CID consisted of one scanning run lasting about 9 min, in which 72 trials were played. At the ontset of each trial, participants saw one of three geometrical cues for 250 ms. Next, they anticipated the appearance of a target square, to which they had to respond with a button press as fast as possible. During target anticipation, a fixation crosshair was shown, and the anticipation period was varied randomly between 2000–2500 ms. Immediately after disappearance of the target, feedback was presented for 1650 ms. Feedback informed participants about whether they had won or lost money during that trial, their total score, and the opponent’s total score (Fig. 4a). “Monetary Gain” cues signaled the possibility of winning € 3 (a circle with three horizontal lines; 32 trials), “Monetary Loss” cues signaled the possibility of losing € −3 (a square with three horizontal lines; 24 trials), and cues representing “no monetary outcome” (€ 0; 12 trials) were denoted by a triangle. The rationale for a larger number of gain trials was that we wanted participants to have the chance to finish the game with a net monetary gain.

To increase the competitiveness of the task, and in line with the strictly competitive task setup we intended to implement, the possible outcomes were arranged in a way that participant and “opponent” were always directly linked to each other’s monetary score. I.e., if participants pressed the button in time before the go cue would disappear from the screen, they would win money, while the (alleged) opponent’s payoff was zero. If they failed to respond fast enough, the opponent received the monetary gain and the participant nothing. If participants pressed the button on time after a loss cue, the opponent would lose money, but not the participant. If they missed, the opposite payoff was the case (Fig. 4b). The main overall goal of the task communicated to participants was to maximize their monetary outcome, and to receive more money than the opponent. Thus, participants were paid the final monetary revenue they had achieved after completing the task. Trial types were pseudorandomly ordered within each run. The display duration of the target cue was adapted to the participant’s performance (within 80–370 ms) to ensure that all participants won in approximately 2/3 of all trial types.

Reaction times were analyzed in SPSS 20.0 (SPSS Inc., Armonk, USA) using a repeated-measures ANOVA with 3 levels for the factor outcome (“Monetary Gain”, “Monetary Loss” and “Non-Monetary Outcome”). Significance was evaluated at P < 0.05. Post-hoc tests with Bonferroni correction for multiple comparisons were applied. Data are reported as means ± SD.

### MRI scanning

MRI scanning was conducted on a 3 Tesla TIM Trio whole body scanner (Siemens, Germany). Participants were scanned using the manufacturer’s 32-channel head coil. Functional images were obtained with a single-shot echo planar imaging (EPI) sequence. The image acquisition parameters were as follows: repetition time (TR) = 1.8 s, echo time (TE) = 38 ms, flip angle (FA) = 90°, 294 whole-brain volumes (matrix size 128 × 128, FoV = 190 × 190 mm^2^, 3 mm slice thickness). For anatomical registration, we obtained high-resolution 3D T1 anatomical images after the fMRI runs (magnetization prepared rapid gradient echo sequence, TR = 2.3 s, TE = 4.21 ms, 1.1 mm slice thickness, 900 ms inversion time, 9° flip angle).

Image analysis was performed using the SPM8 software package (www.fil.ion.ucl.ac.uk/spm) implemented in MATLAB (Mathworks Inc., Natick, USA). Preprocessing included correction for slice timing differences, realignment to the first image to adjust for movement, segmentation, normalization to standard MNI space (at isotropic voxel size), and smoothing with a Gaussian filter (8 mm). The first level (individual subject) analyses were set up using the general linear model approach, with events of interest being modeled by regressors. The fixation cross interval between trials were modeled as an implicit baseline.

**Figure 5:**
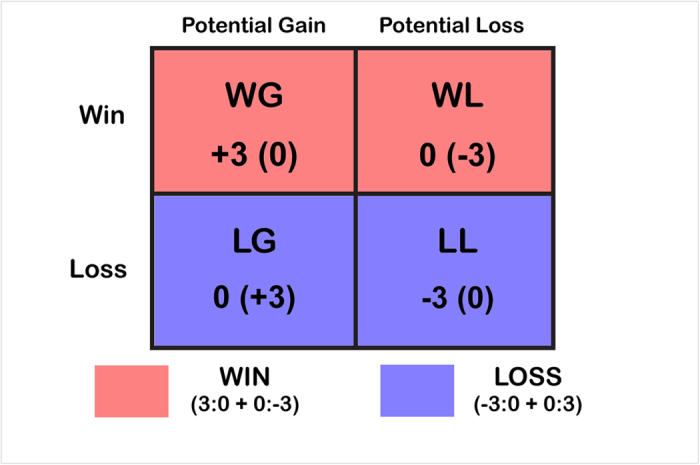
Schematic illustration of the four types of feedback (two types of winning and losing trials, respectively). The red color represents the two conditions of winning, either with Monetary Gain (WG[3:0]) or Avoidance of monetary loss/opponent punishment (WL[0:−3]), while the blue color represents the two types of losing: either with Monetary Loss (LL[−3:0]), or with missed/not acquiring the monetary gain (LG[0:3]). First number in the brackets represents outcomes for participant and second one for the opponent.

The four types of feedback WG[3:0], WL[0:−3], LG[0:3] and LL[−3:0] (two types of wins and losses, in a potential gain or loss framework, respectively) were modeled (Fig. 5).

The anticipation-related responses for all cues were also modeled. Contrast images of these regressors from the first level were then entered into second level random-effects analyses. We used the flexible factorial design option implemented in SPM8 to compare brain activations in response to the different types of feedback.

The contrasts we assessed focused on neural activation differences during the feedback conditions. First, we calculated the contrasts (WG[3:0] + WL[0:−3]) > (GL[0:3] + LL[−3:0]) and (GL[0:3] + LL[−3:0]) > (WG[3:0] + WL[0:−3]) in order to identify the main effect of wins and losses in the game. Secondly, we calculated the interaction (WG[3:0] − LG[0:3]) > (WL[0:−3] − LL[−3:0]) and (WL[0:−3] − LL[−3:0]) > (WG[3:0] − LG[0:3]) in order to see the difference between winning during gain frame trials and winning during loss frame trials. Additionally, we checked separately the contrasts (WG[3:0] > LG[0:3]), (WL[0:−3] > LL[−3:0]) and vice versa, to identify regions specifically involved in winning and losing in gain and loss frame trials.

However, our main interest was to check the contrasts WG[3:0] > WL[−3:0] and WL[0:−3] > LG[0:3] contrasts. The idea behind targeting these specific contrasts was to unveil the neural networks related to self-related or to other-related changes in the monetary scores. For example, the contrast WG[3:0] > LL[−3:0] would inform us about activation related to monetary changes for participants (monetary gain versus monetary loss), while keeping the opponent’s payoff constant (which in both contrasts is zero). Conversely, the contrast WL[0:−3] > LG[0:3] would represent a rather different winning situation, in which while the participant’s monetary payoff was zero, the payoff for the opponent was negative. This contrast WL[0:−3] > LG[0:3] therefore represents a comparison of winning and losing, where we compare the condition “avoidance of monetary loss” (and opponent punishment) versus the condition “missed/not acquiring the monetary gain”.

For all analysis, we used a family-wise error (FWE) correction at the voxel level, at a threshold of P < 0.05 and a cluster extent threshold of 5 voxels, for identifying statistically significantly activated voxels. In some cases where we had strong prior hypotheses, data were also explored at more liberal thresholds (see Results). All results are reported in accordance with recommendations from^50^,^51^.

### Exploratory ROI analysis

For the exploratory analysis of brain activation in regions associated with reward processing and mentalizing, we prepared four regions of interests. Two regions, the right PCC and the right TPJ, were defined as spheres of 8 mm radius, with the ROI center being taken from the peak of activation of the contrast “Mentalizing about Others” versus “Mentalizing about Yourself” from the study of Lombardo *et al.* (8 −58 28 and 60 −60 14, respectively)^38^. Two other ROIs, VMPFC and right nucleus accumbens, were defined in the same way from a meta-analysis^26^. The spheres were based on the peak coordinates found for the analysis of monetary outcome (2 40 −6 and 8 14 −4, respectively).

Mean parameter estimates within four ROI masks were extracted for the contrasts (WG[3:0] > LL[−3:0]) and (WL[0:−3] > LG[0:−3]) from each individual, and entered into statistical analysis. Group differences for each contrast were analyzed in SPSS 20.0 (SPSS Inc., Armonk, USA) using a repeated-measures ANOVA with ROIs as within-subjects factors and Contrast as between-subjects factor. If the sphericity assumption was violated (significant results in Mauchly’s test of sphericity), degrees of freedom were corrected using Greenhouse-Geisser estimates of sphericity. Significance was evaluated at P < 0.05. Post-hoc tests with Bonferroni correction for multiple comparisons were applied.

## Additional Information

**How to cite this article**: Votinov, M. *et al.* Better you lose than I do: neural networks involved in winning and losing in a real time strictly competitive game. *Sci. Rep.* **5**, 11017; doi: 10.1038/srep11017 (2015).

## Figures and Tables

**Table 1:** Cluster list of activation for contrast WG[3:0] > LL[−3:0] (threshold p < 0.05 FWE corrected at voxel level).

**Region**	**L/R**	**Cluster size**	***T***	***x***	***y***	***z***
Putamen	L	423	6.9	−30	−14	0
Caudate	L	s.c	6.6	−20	−3	24
Putamen	L	s.c	6.4	−35	2	5
Caudate	R	127	6.5	21	14	14
Putamen	R	s.c	5.3	27	11	19
Caudate	R	s.c	5.3	18	26	10
Caudate	R	49	6.4	21	−9	29
Caudate	R	s.c	5.3	21	−2	24
Insula	R	89	6.4	38	3	5
Caudate	R	42	6.4	12	11	−10
Putamen	R	s.c	5.8	21	11	−10
Putamen	R	113	6.4	30	−8	5
Putamen	R	s.c	6.2	33	−14	0
Pallidum	R	s.c	5.3	23	−6	0
Parietal Lobe	L	48	6.1	−27	−39	24
Fusiform Gyrus	R	33	5.8	27	−78	−5
Fusiform Gyrus	L	15	5.8	−35	−48	−10
STG	R	20	5.8	45	−33	19
MCC		13	5.7	9	−24	43
STG	L	13	5.6	−59	−11	5
Parietal Lobe	L	20	5.6	−60	−20	43
Occipital		11	5.6	24	−93	14
MCC	L	45	5.6	−3	−9	38
MCC	R	s.c	5.0	5	−15	38
Fusiform Gyrus	R	11	5.5	33	−65	−10
VMPFC		51	5.5	−8	51	0
VMPFC		s.c	5.4	−6	39	−5
SMA	R	6	5.4	8	−14	58
MCC	L	24	5.4	−6	−35	48
Lateral OFC	L	6	5.4	−35	54	−10
Supramarginal Gyrus	R	44	5.3	54	−29	29
Supramarginal Gyrus		s.c	5.1	57	−24	19
Lingual Gyrus	R	6	5.3	23	−66	−5
ACC		11	5.3	−2	17	29
Precentral Gyrus	R	7	5.2	39	−14	43
Temporal Pole		7	5.1	51	−11	14
Temporal Pole		6	5.1	54	2	0

*Note:* L/R = left/right side of the brain; s.c. = sub-cluster; VMPFC = ventromedial prefrontal cortex, ACC = anterior cingulate cortex, PCC = posterior cingulate cortex, SMA = sensorymotor area, MCC = middle cingulate cortex, STG = superior temporal gyrus.

**Table 2:** Cluster list of activation for contrast WL[0:−3] > LG[0:3] (threshold p < 0.05 FWE corrected at voxel level).

**Region**	**L/R**	**Cluster size**	***T***	***x***	***y***	***z***
Putamen	R	608	8.96	26	8	5
Putamen	R		7.48	26	−5	5
Putamen	R		5.90	27	18	−10
Putamen	L	642	8.26	−23	3	5
Putamen	L		8.15	−24	6	−5
Globus Pallidus	L		7.55	−27	−14	5
Thalamus	R	163	6.41	8	−14	0
Caudate	R		6.09	12	−12	10
Hipotalamus	L		6.05	8	−5	−5
Thalamus	L	68	6.25	−8	−14	−5
Midbrain	L		5.00	−8	−23	−5
Occipital	L	151	6.12	−35	−77	−10
IPL	R		5.85	−44	−66	−5
Inferior Occipital Gyrus	R		5.61	−41	−57	−10
Superior Parietal Gyrus	L	27	6.04	−29	−57	43
Inferior Parietal Gyrus	R	50	5.82	38	−41	38
Inferior Parietal Gyrus			5.02	32	−45	43
Middle Occipital Gyrus	L	56	5.60	−26	−74	29
Precuneus	L		5.37	−18	−66	34
IFG	L	16	5.50	−44	2	53
Temporal Lobe	L	12	5.41	−48	−44	5
IFG	L	12	5.28	−44	−2	34
PCC	L	23	5.27	−3	−47	24
IFG	L	10	5.19	−38	17	53
Thalamus		5	5.16	−12	−18	10
Supra Marginal Gyrus		9	5.13	−44	−53	29
Occipital		5	5.09	−24	−95	0
VMPFC		4	5.40	5	−33	−10

*Note:* L/R = left/right side of the brain; s.c. = sub-cluster; IFG = inferior frontal gyrus, IPL = inferior parietal lobule.

**Table 3:** Cluster list of activation for conjunction analysis of the contrasts WG[3:0] > LL[−3:0] and WL[0:−3] > LG[0:3] (threshold p < 0.05 FWE corrected at cluster level).

**Region**	**L/R**	**Cluster size**	***T***	***x***	***y***	***z***
Putamen	L	117	6.70	−30	−14	0
	L	s.c	5.72	−32	−2	0
	L		5.27	−27	−3	10
	R	68	5.98	29	−8	5
	R	22	5.83	21	11	−10
	L	5	5.12	−24	11	0

*Note:* L/R = left/right side of the brain; s.c. = sub-cluster.
